# Public toilets with insufficient ventilation present high cross infection risk

**DOI:** 10.1038/s41598-021-00166-0

**Published:** 2021-10-18

**Authors:** M. C. Jeffrey Lee, K. W. Tham

**Affiliations:** 1 Department of Interior Design, National Taichung University of Science and Technology, Taichung, Taiwan, ROC; 2 Department of the Built Environment, National University of Singapore, Singapore, Singapore

**Keywords:** Environmental sciences, Health care, Engineering

## Abstract

Due to insufficient ventilation, public toilets present high risks for cross-infection. The study investigated 61 public toilets to identify the causes and locations of biological contaminated sources. Airborne and surface bacterial contamination, carbon dioxide concentration, and surface ammonia levels were measured. Both bacterial contamination and CO_2_ are higher in non-ventilated toilets compared to their ventilated counterparts. Bacteria colony forming units (CFUs) in a public toilet with poor ventilation can reach 5 times the number of CFUs outside of the toilet. This suggests that non-ventilated public toilets present a higher risk of cross-infection. Areas near all kinds of sanitary equipment (toilet bowls, squat toilets and urinals) were highly contaminated, indicating that enhanced cleaning regimes are necessary. Further, lidless trash bins present a higher risk as contaminated matter within the trash bins is not inhibited from being released into the environment. Ventilation and cleaning need to be improved to mitigate the risk of cross-infection in public toilets.

## Introduction

The flushing toilet was the invention that ended pandemics in the eighteenth century. However, due to insufficient ventilation, public toilets can become sites for cross-infection. On average, there is high *E. Coli* and some low-risk bacteria shed through feces by each person^1^. Due to insufficient flushing after use, toilets have become a breeding ground for bacteria associated with inadequately removed urine and feces. In addition, some other items like trash cans and miscellaneous items placed in toilets have even facilitated bacteria growth to the extent that hazardous mutations from harmless bacteria have developed to cause some diseases, including dysentery^2^. According to a report published by WHO in March 2020^3^, the SARS-CoV-2 virus may spread through body contact, droplets, aerosols, contaminated materials, feces remnants onto mouth, blood, and from mother to baby, and animals to people^4^. Before using public toilets, there may be a need to remove items of clothing and protective masks, rendering these to become vulnerable moments of potential infection by airborne bioaerosols. Inadequate design of public toilet, water flow and drainage^5^, equipment installation, use, maintenance and management, may result in users being exposed to the environment where infection potentially spread^6^. Thus toilets can become sites for potential cross-infection.

Lee^7^ has investigated 111 public toilets and found that poor designs of public toilets, inappropriate ventilation and equipment result in user complaints. Toilets rank low in architectural consideration in spatial allocation. Poor ventilation and insufficient sunlight can result from this. Poor alignment of sanitary equipment, insufficient or lack of house trap design, broken water seals in the trap due to evaporation, have been documented to facilitate bacteria and viruses in sewage treatment tanks or sewers that subsequently spread to other toilet spaces^8^. Sanitary equipment may be planned without adequate consideration of user behavior; urine may drip or leak; excreta may result in splash or the flush may spray onto the ground or wall near contaminated equipment; gaps between tiles may accumulate dirt and become a good environment for germs to grow. In some countries, due to limited sewer systems, used toilet papers should not be flushed down the toilet but placed in a trash can within the toilet. However, the excreta of carriers left on the toilet paper foster germ growth. The mechanical disturbance caused by the deposition of waste into trash may release aerosols^9^. Under the conditions of insufficient natural ventilation, mechanical ventilation is frequently used to exhaust the indoor bad odors and bio-aerosols but this may be insufficient due to poor air inflow or static pressure of the device.

WHO has proposed an approach to improve indoor ventilation. EU standard EN16798-1 recommends applying 10 L/s/person of ventilation (ventilation rate) in an isolated space or keeping the exhaust fan running continuously to ensure effective ventilation^10^. However, to reduce the spread of the SARS-CoV-2 virus over short distances, the ventilation flow rate should be no less than 3L/s/person^11^. Aerosols emitted by a carrier may remain airborne for a certain period before settling and if the toilets lack proper or sufficient ventilation it becomes a situation for potential cross-infection.

The study investigated and measured current public toilets to identify the causes and locations of biological contaminated sources. It also focuses on exploring the relationship between the ventilation rate and biological contamination.

## Methodology

The study involved randomized sampling in 61 public toilets of high usage (i.e., hospitals, shopping centers, institutes, libraries and transportation stations). In 37 public toilets, the indoor and outdoor bacteria counts were measured. In another 24 public toilets (12 toilets with ventilation and 12 toilets without ventilation respectively) temperature, humidity, carbon dioxide (CO_2_) and airborne bacteria were measured to compare the bacterial contamination levels (measured in units of CFUs) between ventilated and non-ventilated toilets to assess the adequacy of existing ventilation to reduce bacterial exposure.

The inspection method of the indoor concentration of bacteria in the air complies with NIEA E301.10C announced by the Environmental Protection Administration, Executive Yuan, Taiwan^12^. This involved the direct impact of the air sample onto the culture medium which is then incubated at 30 ± 1 °C for 48 ± 2 h, before enumerating the bacteria concentration as colonies forming units per cubic meter (CFUs) of the air. The culture medium is Tryptic Soy Agar prepared in the laboratory. Each liter of the culture medium is composed of Tryptone 15.0 g, Soytone, Thiopeptone or Thiotone 5.0 g, NaCl 5.0 g, and Agar 15.0 g. 40.0 g of these ingredients are dissolved in 1 L of distilled water to form the medium. After heating sterilization, they are cooled and split into around 15 mL of culture medium in a culture plate with a radius of 100 × 15 mm.

**Figure 1:**
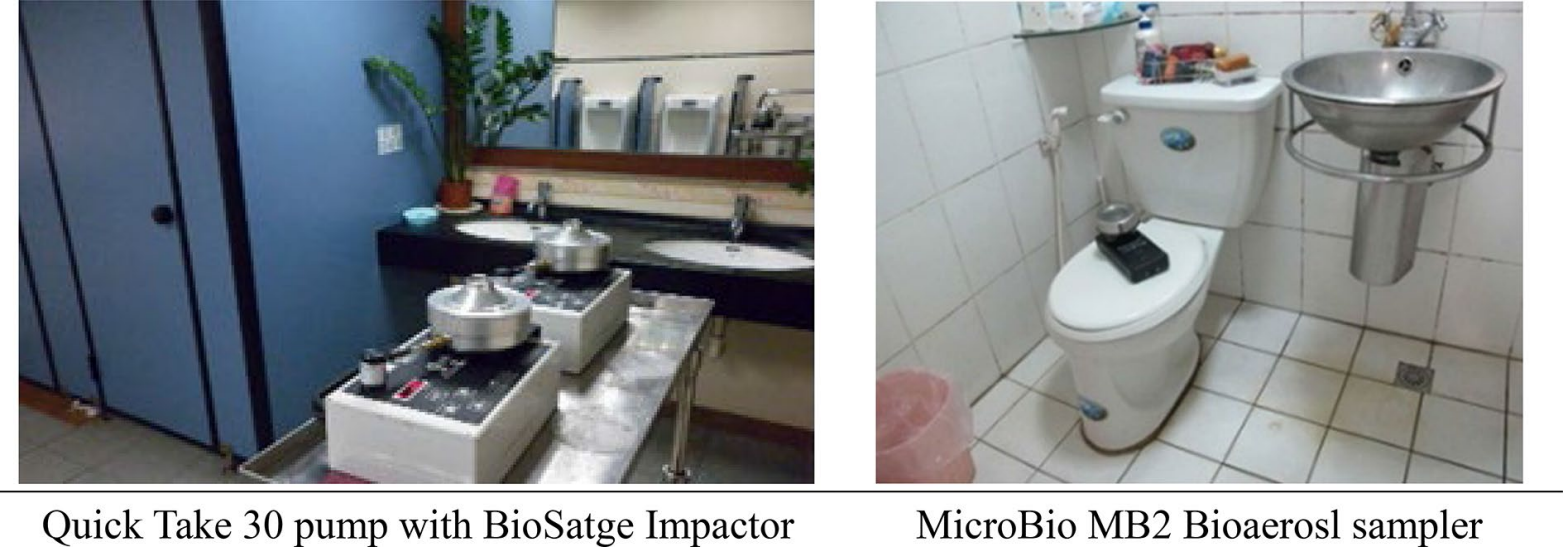
Two types of bioaerosl samplers in the field survey, and the position of the sampling entry for measuring.

Two bio-aerosol impactors were used in the field study to sample 100 L of air for each sampling location: either a BioStage Impactor operated with a Quick Take 30 for 4 min at 28.3 L/min or a MicroBio MB2 Bioaerosol sampler operated for 1 min at 100 L/min. The sampling point is separated from the septum (wall) or the corner with a distance of more than 50 cm. The position of the sampling inlet is placed at a height between 120 and 150 cm above the ground, as shown in Fig. 1. After sampling, the culture plate is capped and sealed, transported to the laboratory and incubated at 30 ± 1 °C for 48 ± 2 h, before enumerating the bacterial CFUs.

Surface samples of bacteria are retrieved in the immediate area of the sanitary equipment to assess the potential contamination due to the impact of splashing. Chi^2^ and Chu^13^ showed that bacteria may adhere to the surfaces and grow. The most common area for stain is the toilet itself and the floor surrounding the toilet. Stain accumulates easily and provides a good environment for bacteria growth if not cleaned properly. This study explores the severity of surface bacterial contamination attributable to splashing by user behaviour (standing urine, non-water flow area, flushing the toilet without cover) and the locations of stain accumulation.

Surface sampling of different types of toilets was conducted, including common toilets (WC), squat toilets (ST), urinal (U) and trash cans (TB)^14^. Water outlets tend to have residual water stains due to long-term water flow, as do the area surrounding the wetted surface in the toilet. Hence, this area has been identified as the first sampling point (WC1). The margin around the toilet itself (WC2 and WC3) is the area usually splashed and dripped on so they are also sampling locations. The inner parts of the toilet are not easily cleaned so it may lead to stain accumulation and the growth of bacteria in the long term. This is sampling point WC4. The lining between the floor and the toilet is usually completed using porous materials such as cement mortar, which is frequently ignored during cleaning. Bacteria and fungi often grow here so this is added to the sample as WC5. In general, users flush the toilet without closing the lid so bacteria inside the toilet is easily released into the space, with frequent splashing onto the floor. This area is WC6, as noted in Table 1. The periphery of the squat toilet is prone to stain accumulation and causes bacteria to grow. This spot is assigned as the ST1 sampling point. Where the squat toilet joins the floor is commonly constructed by porous cement mortar so bacteria and fungi grow easily there as well; this is ST2. Flushing of the toilet often results in splashing onto the surrounding floor and is identified as ST3.

**Table 1:**
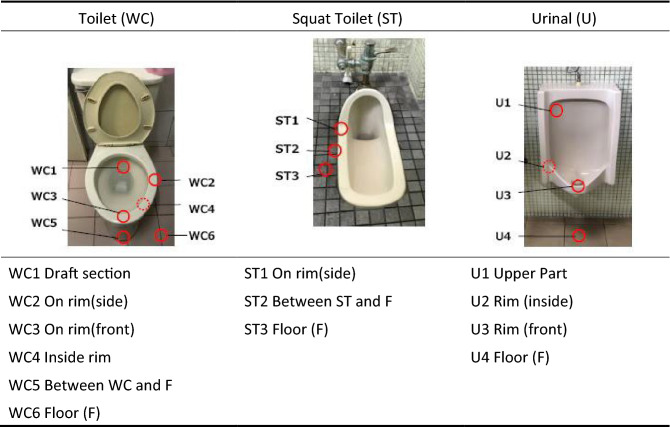
The sampling point of bacteria on the toilets.

The water outlet of the urinal may have some residual fluid due to imbalanced water flow or poor cleaning; so this is another place that bacteria can grow. The outlet is marked as sampling point U1. The inner peripheral surface of the urinal is not easy to clean thoroughly and urine stain can accumulate; this is marked as U2. The protruding part in front of the urinal accumulates urine and can become an area for bacteria growth and virus accumulation if not sufficiently cleaned—this is marked as U3. Furthermore, if users have benign prostatic hyperplasia or undesired urination habits, urine may drip to the floor; this is sampling point U4.

**Figure 2:**
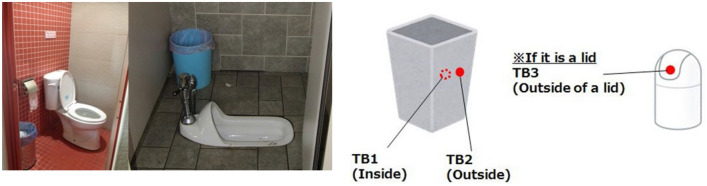
The sampling point of the trash can.

In some countries, due to bad sewers or poor sewer system designs, used toilet papers may not be thrown into the toilet to prevent pipelines and managing equipment from becoming obstructed. Sanitary materials or waste materials are also not permitted to be discharged into the flushing system. There are trash cans placed to the side of the toilet to discard toilet papers and sanitary waste. However, wastes with dirty substances expose the trash box to bacteria and viruses; it essentially becomes an incubator for microorganisms to grow, especially trash cans without lids. Inside the trash can is the most direct spot to encounter contaminated substances—this is designated as sampling point TB1. Sampling point TB2 is the corresponding spot as TB1 outside of the trash can; and their measurements provide a comparison of bacterial contamination. Trash can designs are quite diverse, and may be are classified into two types: with lid and without lid. Trash cans with a lid have one more sampling point, marked as TB3 in Fig. 2.

**Figure 3:**
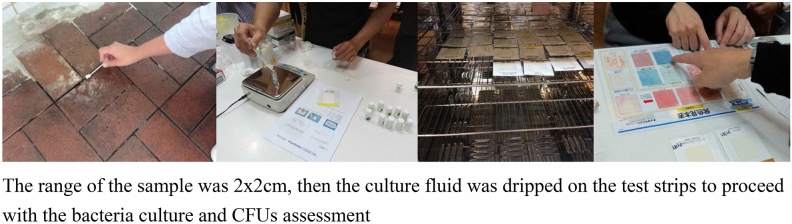
The bacteria culture process and CFUs assessment.

As indicated by Su^15^, most of the bacteria found in the toilet are *E. Coli*. spp. and Staphylococcus spp. To check the locations and quantities of these two bacteria in the toilet, sampling strips are used to retrieve *E. Coli*. (MC-Media Pad “EC”) and MC Media Pad “Aerobic count” was used as a bacteria test. For all sampling locations, culture swabs over a 2 × 2 cm wiping square were performed. The sampling swab was then inserted into the culture medium and the lid closed on the sampling bottle. The bottles of samples were stored at a temperate of under 5 °C and cultured for 12 h. The sampling swab was then taken out of the culture medium and sampling fluid was dripped on the bacteria test strips which were subsequently placed in an oven heated to 35 °C then removed after culturing for 24 h for observation. The strips were then classified based on samples from different sampling toilets and locations, then recorded for bacteria distribution volume based on the bacteria changes on the strips, shown in Fig. 3. The colors shown on the strips are used to evaluate the bacterial contamination (CFUs), as shown at the bottom of Fig. 3.

**Figure 4:**
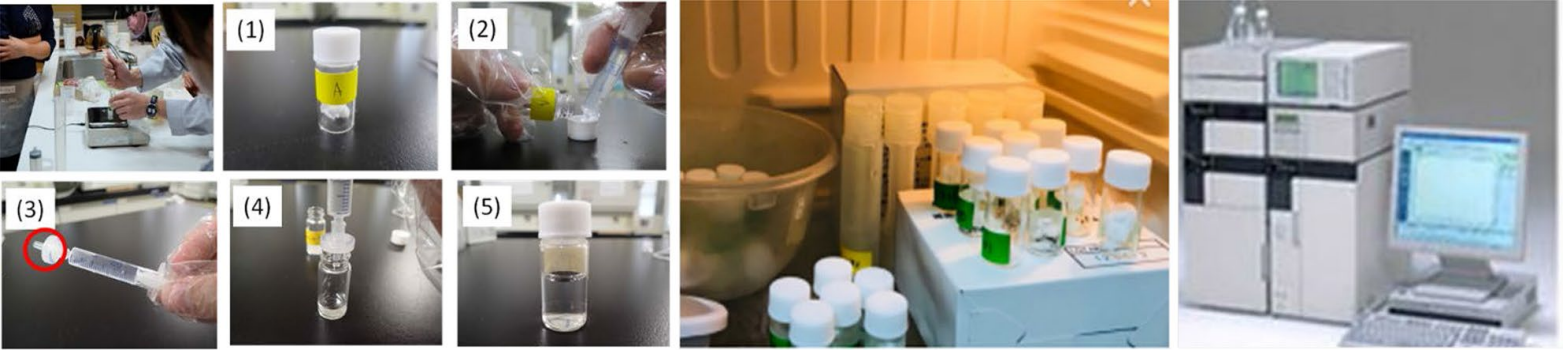
Sample of stain for the culture, NH_3_ and Na content analysis.

In addition, sampling and analysis of the stain collected from those areas were identified based on the locations of stain accumulation and the relationship between the soiled material compositions and the bacteria quantities. The area was wiped with either dry or wet cotton balls and placed in the same glass bottle. 5 ml of distilled water was added and the bottle was placed in the refrigerator below 5 °C to culture for 12 h. The 5 ml of culture fluid was aspirated with a syringe and a filter placed in front of it to inject the culture fluid into a new glass bottle. The extracted culture fluid was added to the liquid chromatography LC/MS to analyze the NH_3_ and Na contents of the stain in the culture fluid, shown in Fig. 4.

## Results and discussion

### CFUs in the air

The randomly selected 37 public toilets with different sizes of space and users are located in 5 different types of venues: 10 in shopping malls (M), 8 in hospitals (H), 8 in offices (O), 7 in the libraries (L), and 4 in the stations (S). Most of the public toilets are not equipped with a ventilation system: ventilation-equipped public toilets constitute 22% and non-ventilation-equipped public toilets 78%.

**Figure 5:**
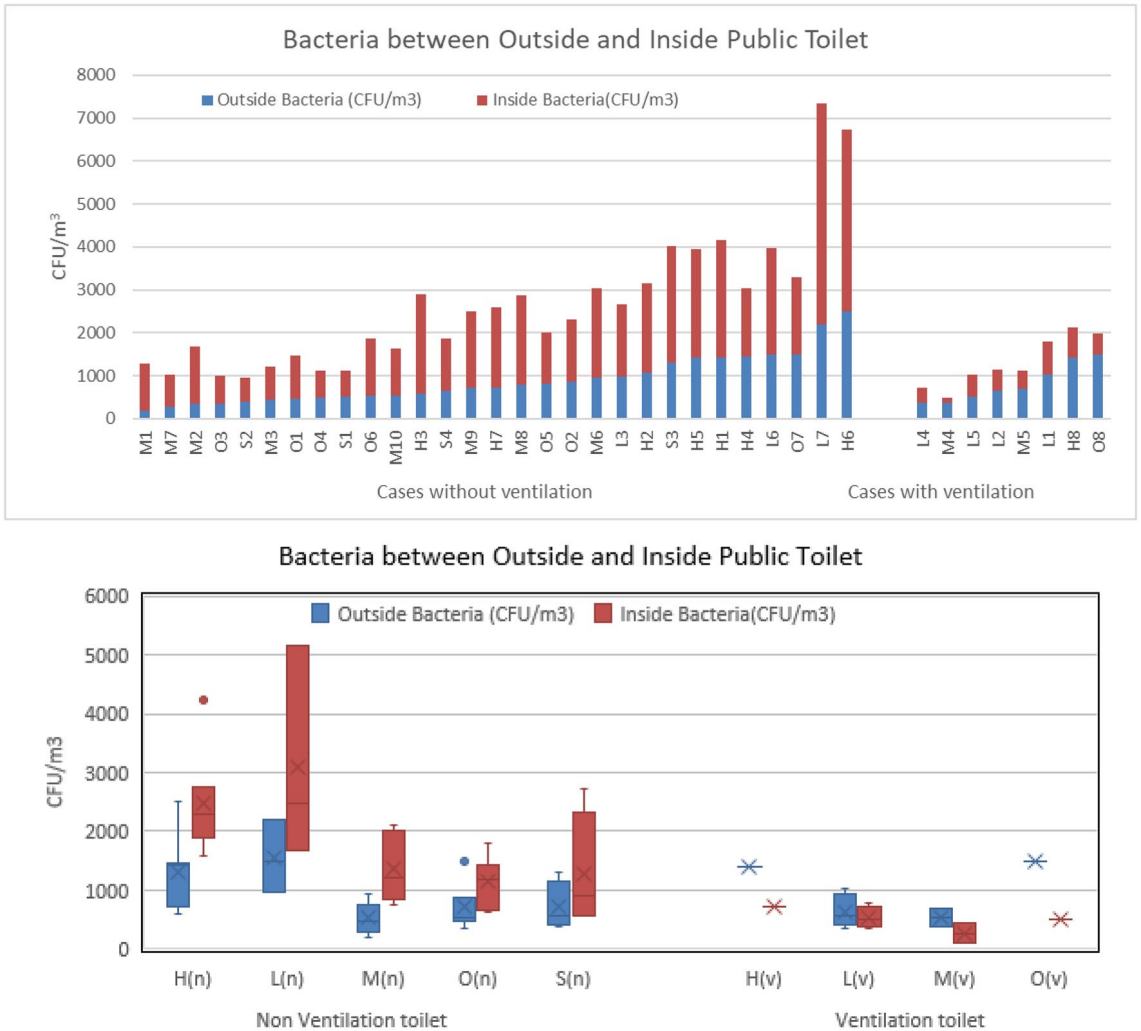
The collected CFUs inside and outside of public toilets with ventilation and without ventilation.

Figure 5 compares the bacteria colonies inside and outside of public toilets. The bacterial contamination (CFUs) cultured from the indoor samples collected from ventilation-equipped public toilets were lower than outdoor levels (CFUs). In contrast, the CFUs of indoor samples from public toilets without ventilation were between 1.5 and 5 times higher than the CFUs of outdoor samples. If the carriers entered those public toilets, the viruses produced by the carriers could persist in the exhaled aerosols, in the flushing fluid (potentially containing fragments of excreta) splashed onto the floor or the bio-waste from lid-less trash cans. These remain in the public toilets, potentially presenting an exposure risk to the next users. In public toilets inside hospitals and few libraries, the CFUs were significantly higher than any other venues, as shown in the cultured results in Fig. 5. This finding is similar to Chen^16^ who highlighted high cross-infection risk in hospitals.

**Figure 6:**
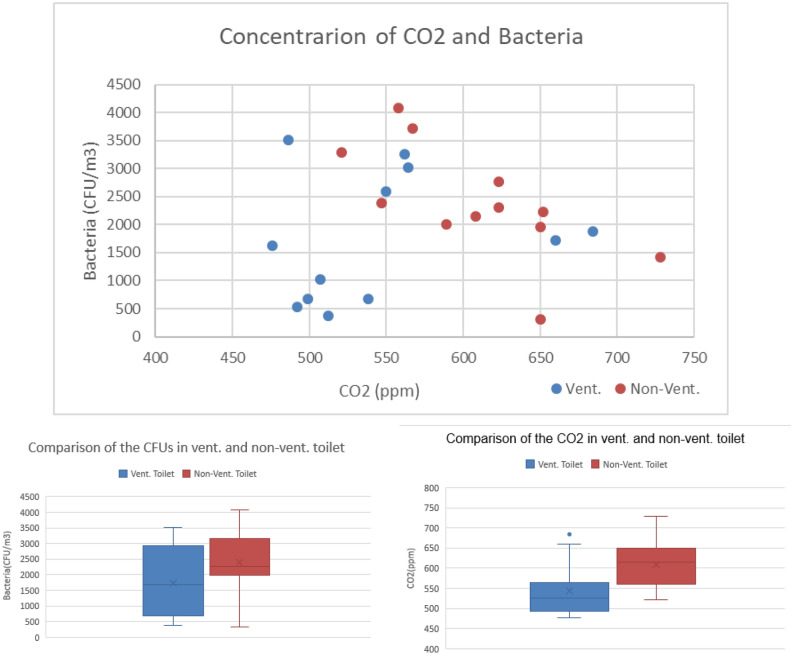
Comparison of the CFUs in ventilation toilet and non-ventilation toilet.

No environmental sampling was conducted in these 37 public toilets, and the data from 24 other public toilets which included measurements of carbon dioxide, temperature and humidity in addition to bacterial contamination was analyzed for the correlation between CO_2_ and bacterial contamination. These comprise 12 ventilation-equipped toilets and 12 non-ventilation-equipped toilets. The boxplot of bacterial contamination and CO_2_ is shown in Fig. 6. Though not statistically significant, there is an observable trend. Both bacterial contamination and CO_2_ are higher in non-ventilated toilets compared to their ventilated counterparts. This suggests that non-ventilation-equipped public toilets have higher bacteria concentration and presents a higher risk of cross-infection.

### The highly contaminated area near toilets

Data from the sampled 61 public toilets showed that the areas near all kinds of sanitary equipment were highly contaminated (more red dots on the test strips means the higher CFUs). Inside the toilets, bacteria can be swept away with a flush. In contrast, if urine stains accumulate around the floor due to undesirable user behavior, the floor surrounding the toilet becomes contaminated and the best place for bacteria to grow. The worst area for contamination is the lining between the toilet and the floor (WC5) as well as the floor surrounding the toilet (WC6). Due to the posture required to use a squat toilet, the sampling points were different from a regular toilet. The sampling locations still focus on toilets and the surrounding area and floor. As the bacteria counts around the toilet and the surrounding floor were high, it shows that different user behaviors may lead to different contaminated ranges and levels. The most contaminated area of the squat toilet was the same as a regular toilet; the lining between the toilet and the floor (ST2) and the floor around the toilet (ST3). As the water flows inside the urinal when flushed, the content of the bacteria was lower than on the floor beneath it. The most contaminated area of the urinal was the floor beneath the urinal (U4), as shown in Table 2.

**Table 2:**
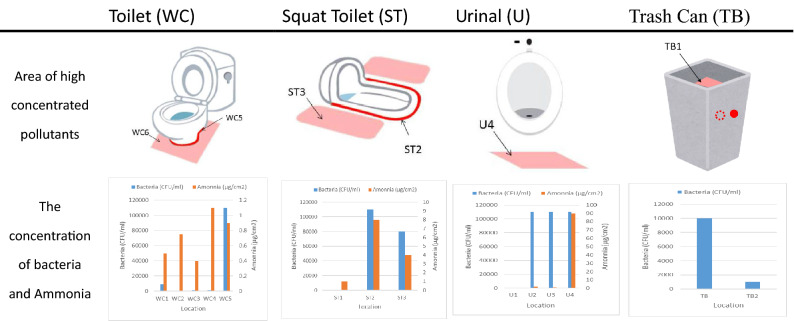
Highly contaminated areas near toilets.

The levels of stain were determined from the ammonia content data of the samples, then the average concentration of all samples was taken. The ammonia (an ingredient of urine) content of the stain is higher in samples from the area near the toilet. The highest concentration was from the samples collected from the floor around the toilet (WC6). The second highest was from the lining between the toilet and the floor (WC5). In the squat toilet, the highest concentration was from the lining between the toilet and the floor (ST2), and the second-highest was the ground surrounding the toilet (ST3). As for the urinal, the highest level was found on the floor (U4) and levels were not low in other locations as shown in Table 2.

The sampling points inside and outside of the trash can showed the bacteria inside the trash can is higher than it outside the trash can. Most trash cans do not have a lid to facilitate the throwing of waste into the trash can. Bacteria growth on the toilet paper increases the bioaerosol load and raises the risk of cross-infection.

Locations of highest bacterial contamination are pervasive around the sanitary equipment; thus removing the biological contaminating substances from those locations is the key to reducing cross-infection risk. For example, high-frequency cleaning of such locations shown in Table 2 is essential to reducing the breeding the bacteria. The use of strong alkaline detergent impedes *E. Coli*. growth on the toilet. The application of suitable detergents may inhibit the growth of bacteria and viruses on the floor areas around the toilet as these are heavily contaminated zones. Furthermore, if the humidity and biological aerosols inside the toilet can be effectively removed^17^ and UV-C light used while no occupants inside the public toilet in combination^18^, biological contamination sources could effectively be removed as well.

## Conclusion

Bacterial contamination results of the surface sample swabs near sanitary equipment in public toilets indicate that the concentration of organisms is a serious issue. These surfaces are close to users of public toilets, and together with bacteria aerosolized from various activities including operation of sanitary equipment, present channels of transmission of viruses. If the building materials and trash cans without lids are not effectively resistant to bacteria, or if there is no sterilization equipment or sufficient ventilation in public toilets, the bacterial load in public toilets exceed that of the outdoors by factors between 1.5 and 5 times. In spaces with poor ventilation, bacteria will remain in the toilets and grow to become a high risk of cross-infection. As it would take considerable efforts and investment to upgrade the building materials and equipment of public toilets, better ventilation should be considered to reduce the risk of cross-infection.
